# An Interactive Channel Model of the Basal Ganglia: Bifurcation Analysis Under Healthy and Parkinsonian Conditions

**DOI:** 10.1186/2190-8567-3-14

**Published:** 2013-08-14

**Authors:** Robert Merrison-Hort, Nada Yousif, Felix Njap, Ulrich G Hofmann, Oleksandr Burylko, Roman Borisyuk

**Affiliations:** 1 School of Computing & Mathematics, Plymouth University, Drake Circus, Plymouth, PL4 8AA, UK; 2 Neuromodulation Group, Division of Brain Sciences, Faculty of Medicine, Imperial College London, London, UK; 3 Graduate School for Computing in Medicine and Life Sciences, University of Lübeck, Lübeck, Germany; 4 Department for Neurosurgery, Albert-Ludwigs-University Freiburg, 79108, Freiburg, Germany; 5 Institute of Mathematics, National Academy of Sciences of Ukraine, 3 Tereshchenkivska Street, Kyiv, 01601, Ukraine; 6 Institute of Mathematical Problems in Biology, Russian Academy of Sciences, Pushchino, Russia

**Keywords:** Parkinson’s disease, Mean-field model, Bifurcation analysis, Beta oscillations, Subthalamic nucleus, Globus pallidus, Wilson–Cowan equations

## Abstract

Oscillations in the basal ganglia are an active area of research and have been shown to relate to the hypokinetic motor symptoms of Parkinson’s disease. We study oscillations in a multi-channel mean field model, where each channel consists of an interconnected pair of subthalamic nucleus and globus pallidus sub-populations.

To study how the channels interact, we perform two-dimensional bifurcation analysis of a model of an individual channel, which reveals the critical boundaries in parameter space that separate different dynamical modes; these modes include steady-state, oscillatory, and bi-stable behaviour. Without self-excitation in the subthalamic nucleus a single channel cannot generate oscillations, yet there is little experimental evidence for such self-excitation. Our results show that the interactive channel model with coupling via pallidal sub-populations demonstrates robust oscillatory behaviour without subthalamic self-excitation, provided the coupling is sufficiently strong. We study the model under healthy and Parkinsonian conditions and demonstrate that it exhibits oscillations for a much wider range of parameters in the Parkinsonian case. In the discussion, we show how our results compare with experimental findings and discuss their possible physiological interpretation. For example, experiments have found that increased lateral coupling in the rat basal ganglia is correlated with oscillations under Parkinsonian conditions.

## 1 Introduction

The basal ganglia are a group of densely interconnected subcortical nuclei comprising of the striatum, globus pallidus, subthalamic nucleus (STN), and substantia nigra. Cortical projections to the ventral striatum and STN provide input to the basal ganglia from almost all areas of the cortex [1-3]. In primates efferent output from the basal ganglia innervates ascending and descending neurons in the thalamus and brainstem, via the internal segment of the globus pallidus (GPi) and the substantia nigra pars reticulata (SNr) [4,5]. The basal ganglia therefore appear to be in a key position to modulate the flow of information along motor and sensory pathways. 

Parkinson’s disease is primarily a disease of the basal ganglia. Its main pathophysiological feature is the death of the neurons in the substantia nigra pars compacta (SNc) that provide widespread dopaminergic innervation to the other basal ganglia nuclei [6]. Electrical recordings from animal models of Parkinson’s disease and patients undergoing functional neurosurgery have revealed several characteristics of the electrical activity in the Parkinsonian basal ganglia that presumably arise as a result of this loss of dopaminergic input. Perhaps the most well studied of these pathological features is a marked increase in the degree of widespread synchronised oscillatory activity within the STN and GPi. This increased synchrony is shown by an increase in spectral power of the local field potential (LFP) signal recorded from these nuclei, particularly within the so-called *β* frequency band (8–30 Hz) [7]. LFP power in this range decreases when patients are taking the dopamine prodrug l-DOPA and has been shown to be positively correlated with the severity of the main hypokinetic motor symptoms of Parkinson’s disease: bradykinesia and rigidity [8]. Although in general LFPs appear to better represent subthreshold synaptic currents rather than widespread spiking activity [9], several studies have found that (in the STN at least) the LFP signal is indeed linked to the activity of local neurons [10-12]. 

There is some experimental evidence that supports the idea that excessive levels of synchronous *β* activity are the causal basis for bradykinesia and rigidity [13]. Macro-electrode stimulation of the STN at 20 Hz reduces the speed of movement during a finger tapping task [14] and slows force development in a grip task [15]. Exactly how synchronous *β* activity could have an anti-kinetic effect remains to be seen. One theory considers the basal ganglia as one or more information channels in which the available bandwidth corresponds to the degree of independence between neurons [16]. In the pathological case where many neurons have become entrained to fire synchronously in time with a particular rhythm then the ability for the basal ganglia to convey meaningful information would clearly be limited. An alternative hypothesis holds that *β* oscillations are a globally coherent signal that correspond to tonic maintenance of the current pose [17]. This is supported by evidence from monkeys [18] and humans [19] that shows that during tonic muscle activity there are widespread synchronous *β* oscillations in both the central and peripheral nervous system. It is suggested that this oscillatory activity may be subject to modulation in the basal ganglia, with dopamine acting as an indicator of movement-related stimuli that reduces the level of synchronous *β* activity [20]. 

Understanding the nature of abnormal *β* oscillations in Parkinson’s disease may lead to new treatments and, more generally, insights into the motor functions of the basal ganglia. Two important questions are where the oscillations arise and the mechanism by which they are generated. While it is possible that they are of cortical origin [21], Parkinson’s disease primarily affects the nuclei of the basal ganglia so it seems plausible that these nuclei are somehow involved in the generation of *β* rhythms. Two potential sources within the basal ganglia that have been suggested by experimental and theoretical work are the striatum and the reciprocally connected neurons of the STN and external segment of the globus pallidus (GPe). LFP recordings from healthy monkeys show transient *β* oscillations that are synchronous across large areas of the striatum [22]. In [23], McCarthy et al. develop a computational model of a network of striatal medium spiny neurons that shows a peak in the *β* power of a simulated LFP that increases under reduced dopamine conditions. The basis for these oscillations in their model is a non-inactivating potassium current known as “M-current”. A key prediction of the model, that increased striatal acetylcholine levels will increase *β* power, was verified in rodent experiments as part of the same study.

The reciprocally connected neurons of the GPe and STN have been more extensively studied as a possible source of *β* oscillations than the striatum. Intra-cellular tracing studies suggest that both the inhibitory GABAergic projection from GPe to STN and the excitatory glutamatergic projection from STN to GPe show a great degree of spatial selectivity, with individual groups of pallidal neurons projecting to individual groups of subthalamic neurons, which in turn project back to their afferent pallidal neurons [24]. Since STN neurons are capable of rebound firing upon release from GABA-mediated hyperpolarization [25], this arrangement suggests that reciprocally connected groups of STN-GPe neurons may be able to act as pacemaker circuits. In vitro co-cultures of cortical, striatal, pallidal, and subthalamic cells show that neurons in the GPe-STN circuits are indeed capable of generating oscillatory firing patterns in the absence in rhythmic inputs [26]. Experiments in Parkinsonian primates in which synaptic connections in the basal ganglia were selectively blocked demonstrated that *β* oscillations were dependent on glutamatergic input to the STN and the reciprocal connections between the STN and GPe [27]. 

Computational and mathematical modelling has also demonstrated that the GPe and STN are capable of acting as a pacemaker circuit. The detailed conductance-based models of Terman et al. [28] show that GPe-STN networks with various topologies are capable of producing a wide range of different activity patterns, including transient oscillations. An average firing rate model of a coupled pair of GPe and STN populations suggests that robust *β*-band oscillatory activity is possible provided that self-excitation within the STN population exceeds a certain level [29]. It seems unlikely, however, that STN neurons exert any excitatory influence over other STN neurons since there is no evidence of local axon collaterals or gap junctions within the nucleus [30,31]. A similar modelling study has demonstrated that with the addition of synaptic delays, coupled GPe-STN populations can generate *β* oscillations without any STN self-excitation [32]. 

The aim of this work is to extend the population-level models of [29] and [32] to investigate the behaviour when multiple interactive groups (or “channels”) of GPe and STN neurons are present. This is based on the idea that information flows through the basal ganglia in circuits that remain largely segregated [3,33,34]. It should be noted that it is unclear from the current biological data to what level of representation this segregation is maintained and, therefore, whether channels correspond to body regions, limbs, individual muscles or even particular motor actions. In general, we do not assume any level of representation and simply seek to identify what dynamics are possible in a system of coupled parallel channels. We initially study a single isolated channel and use two-parametric bifurcation analysis to find the critical boundaries in parameter space that separate regions of different dynamics. This bifurcation analysis provides useful guidance for the study of the collective behaviour of locally coupled channels (arranged in either a circle or line topology); in particular it suggests parameter values that correspond to oscillatory dynamics. Additionally, while [29] studied the dynamics of the system under changing levels of excitatory and inhibitory striatal GPe input, the neurons that project from striatum to GPe are usually silent [35]. We investigate the possibility that the direct cortical projection to the STN (the “hyper-direct” pathway) plays an important role in modulating pallidal and subthalamic activity [36]. 

Section 2.1 will describe the model and introduce its equations and parameters. In Sect. 3, we will present the results of bifurcation analysis and numerical simulations of the model in the case of a single uncoupled channel. These results are used to inform the analysis of the locally coupled model, which we will present in Sect. 4. Section 5 will discuss a possible physiological interpretation of our results and compare them with previous experimental and theoretical studies.

## 2 Methods

### 2.1 Model Description

The model consists of 2*N* coupled non-linear differential equations, where *N* is the number of channels being modelled:

(1) $$\tau_{s}{\dot{x}}_{i}=-x_{i}+Z_{s}(w_{ss}x_{i}-w_{gs}y_{i}+I)$$

(2) $$\tau_{g}{\dot{y}}_{i}=-y_{i}+Z_{g}\left(-w_{gg}y_{i}+w_{sg}x_{i}-\alpha w_{gg}\sum_{j\in L_{i}}y_{j}\right),\;i=1,2,\ldots ,N$$

These equations are based on those developed by Wilson and Cowan [37]. The time-dependent variables $x_{i}$ and $y_{i}$ represent the mean field activity of the excitatory STN subpopulation and inhibitory GPe subpopulation of channel *i*, respectively. Taken together the equations represent a pair of reciprocally connected STN-GPe sub-populations corresponding to one of many hypothesised basal ganglia information channels [34]. The connection strength parameters ($w_{ss}$, $w_{sg}$, $w_{gg}$, and $w_{gs}$) are non-negative and represent the strength of synaptic connectivity within and between the populations, where $w_{pq}$ is the connection strength from population *p* to population *q* (e.g. $w_{sg}$ is the synaptic connectivity from STN to GPe). $\tau_{s}$ and $\tau_{g}$ represent the average membrane time constants of neurons in the two populations, while *I* represents a constant level of cortical excitation of the STN (the hyper-direct pathway). For simplicity, this study is restricted to the case when there is the same degree of constant cortical input to each of the channels.

Connections between the channels take the form of lateral inhibition between GPe sub-populations. The strength of this lateral coupling is taken to be a proportion *α* of the coupling strength within GPe sub-populations ($w_{gg}$), where α≥0. Different connection schemes are possible and are specified by the term $L_{i}$ in Eq. (2). For a given channel *i*, $L_{i}$ is a set of indexes that specifies which channels the GPe subpopulation receives inhibition from. In this study, we consider only local connections to immediate neighbours, with two different arrangements of channels: on a line (Eq. (3)) and on a circle (Eq. (4)).

(3) $$L_{i}=\{\begin{array}{cc} \left\{i-1,i+1\right\} & 1<i<N\\ \left\{i+1\right\} & i=1\\ \left\{i-1\right\} & i=N \end{array}$$

(4) $$L_{i}=\{\begin{array}{cc} \left\{i-1,i+1\right\} & 1<i<N\\ \left\{i+1,N\right\} & i=1\\ \left\{i-1,1\right\} & i=N \end{array}$$

The system is non-linear due to the functions $Z_{s}(\cdot )$ and $Z_{g}(\cdot )$, which represent how different levels of synaptic input influence the activity of the population. The functions are sigmoidal in shape and are described by Eq. (5):

(5) $$Z_{j}(x)=\frac{1}{1+\exp(-a_{j}(x-\theta_{j}))}-\frac{1}{1+\exp(a_{j}\theta_{j})}$$

**Fig. 1:**
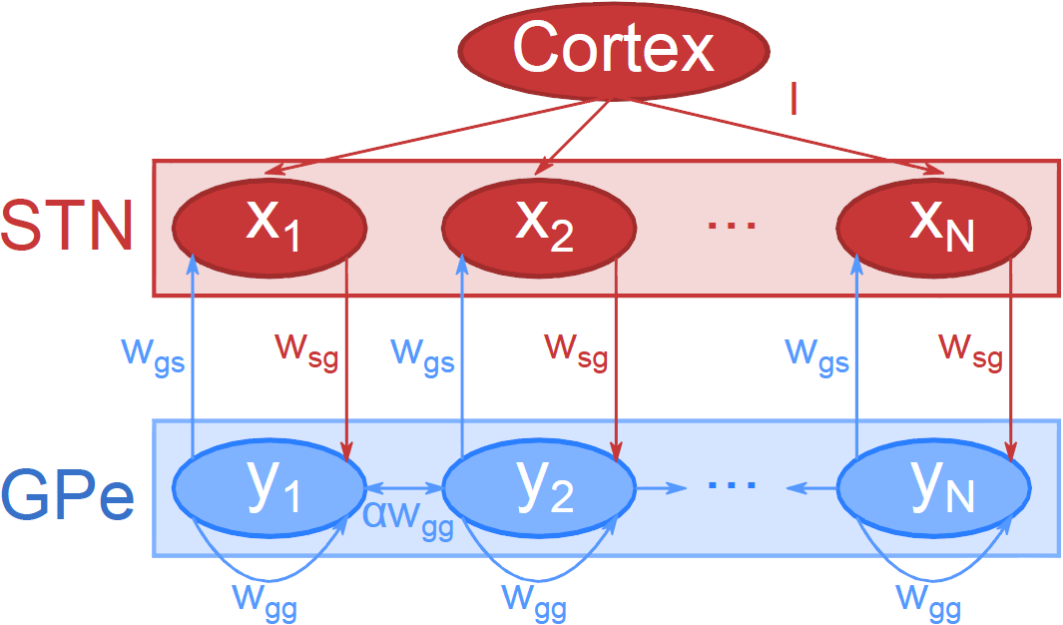
Model schematic. Schematic diagram showing the system arranged in a line topography, including the excitatory STN sub-populations, the inhibitory GPe sub-populations, and the connections between them. *Red* represents excitatory sub-populations and connections; *blue* represents inhibitory sub-populations and connections

Here, j=s or *g*. This adds four new parameters, $a_{s}$, $a_{g}$, $\theta_{s}$, and $\theta_{g}$, which represent the maximum slope of the sigmoid and its position on the horizontal axis respectively, for STN and GPe sub-populations. The constant term that is subtracted in Eq. (5) is used in the Wilson–Cowan formalism to ensure that $Z_{j}(0)=0$, which means that when a subpopulation receives no inputs its activity tends to a single stable fixed point [37]. The model is summarised in Fig. 1. Note that the cortex is not modelled as a population, it simply provides a constant level of input to each STN subpopulation. 

### 2.2 Parameter Values

For the values of $a_{s}$, $a_{g}$, $\theta_{s}$, and $\theta_{g}$ we use the typical values for excitatory and inhibitory sub-populations specified by Wilson and Cowan [37]. For the remaining fixed parameters, the values determined by Holgado et al. [32] are used. Due to a lack of experimentally determined electrical characteristics of neurons in the primate basal ganglia, the membrane time constants used are those from rodent studies. It should be noted, however, that neurons in the rodent globus pallidus appear to vary widely in their electrical characteristics, and the value for $\tau_{g}$ used here (from [32]) lies below the range of values estimated by some experimental studies (see, e.g. [38]). 

Two sets of values for the connection strengths are used, which will be termed the “healthy” and “Parkinsonian” parameters. Holgado et al. determined these parameters on the basis of previously published experimental recordings of unit activity from the STN and GPe of monkeys. Recordings were used from both healthy animals and animals that were rendered Parkinsonian via 1-methyl-4-phenyl-1,2,3,6-tetrahydropyridine (MPTP) lesioning. In each case, the recorded firing rate under a variety of conditions (normal, transmitter block, current injection) was compared to the firing rate predicted by their model under the same (simulated) conditions, and a genetic algorithm was used in order to find two sets of connection strengths that best fit the data. The parameter fitting that Holgado et al. performed suggested that all connections became stronger under Parkinsonian conditions, and they cite several experimental results that support this increase, including the presence of $D_{2}$ receptors in the STN [39] and GPe [40,41] and the enhanced effect of GABA on STN neurons [42,43] and glutamate on GPe neurons [41,44] when dopamine is reduced. We note that MPTP lesioning represents chronic dopamine depletion, which is the condition under which synchronised *β* activity is seen in experiments. In Holgado et al.’s model, the system has only steady-state behaviour when the healthy parameters are used, but linearly scaling the parameters towards the Parkinsonian values causes stable *β* oscillations to appear. See Table 1 for fixed parameter values. It should be noted that the equations in Holgado et al.’s model directly represent the average unit firing rate of the populations. In this regime, each weight parameter has a direct physical meaning: it represents how many spikes/s the target population increases (or decreases) by when the source population’s firing rate increases (or decreases) by 1 spike/s. Our model does not directly represent the firing rates of populations, and so the parameters should therefore be interpreted as representing the general relative strengths of synaptic connections. 

**Table 1:** Fixed parameter values for the healthy and Parkinsonian conditions

	Healthy	Parkinsonian
$w_{gg}$	6.6	12.3
$w_{gs}$	1.12	10.7
$w_{sg}$	19.0	20.0
$\tau_{s}$	6 ms	
$\tau_{g}$	14 ms	
$a_{s}$	4	
$\theta_{s}$	1.3	
$a_{g}$	3.7	
$\theta_{g}$	2	

### 2.3 Technical Details

For qualitative investigation of the isolated channel model, we used the software package XPPAUT [45] with the default integrator, a fourth-order Runge–Kutta method and a fixed step size of 0.5 ms. Numerical continuation in the isolated channel model was carried out using XPPAUT and LOCBIF [46]. In a few cases, numerical continuation failed to compute some parts of the 2D bifurcation diagram and in these cases the analysis was performed by fixing one parameter and observing the changing dynamics as the other was carefully varied. Qualitative investigation of the coupled channels model was done using XPPAUT and associated XPPy Python interface [47]. Numerical continuation of this system was carried out using CONTENT [48]. 

The frequency visualisation plots were computed using XPPAUT and XPPy for the numerical simulation and the FFT routine from the SciPy library [49]. When calculating the FFT the total integration time was 2.048 s, but only the second half of the integration output was passed to the FFT routine to try to ensure that the trajectory was close enough to the stable limit cycle for only truly oscillatory activity to be included. This gave the FFT output a range of 0–1024 Hz across 1024 bins. This was repeated five times for each parameter pair, with random initial conditions. The frequency and amplitude of the most powerful FFT bin over the ten runs were recorded and plotted. 

## 3 Isolated Channel Model

This section will consider the simplified system that is obtained by setting α=0. This condition corresponds to the case where neurons from each STN and GPe subpopulation never make synapses onto neurons outside their own channel. The detailed study of a single element enables us to understand some aspects of the dynamics in the system of interactive channels. For example, the boundaries of oscillatory regimes in the 2D bifurcation diagram allow estimation of the level of input channels must receive from the cortex and their neighbours in order to give oscillatory dynamics.

Since it is only necessary to consider two equations in this reduced model, bifurcation analysis can be used to completely understand the different dynamical regimes that are possible within a single channel. We consider the bifurcations of the system under variation of the following two parameters: 

• The level of cortical input to the STN (*I*). There are two major pathways by which cortical input reaches the basal ganglia: one via the striatum and one projecting directly to the STN. Striatal projection neurons fire very infrequently during periods of rest, so the system’s behaviour in response to varying levels of steady-state input via the cortico-subthalamic “hyper-direct” pathway is studied.

• The amount of self-excitation within the STN ($w_{ss}$). The work of Gillies et al. [29] suggests that there must be some ability for STN neurons to provide excitation to other STN neurons in order for the STN-GPe network to exhibit oscillations. Since the biological plausibility of this is contentious, bifurcation analysis is used to determine how much STN self-excitation is required for oscillations and how this depends on the level of hyper-direct input. It is also useful to study the behaviour of the isolated channel model under variation of $w_{ss}$ because the laterally coupled GPe sub-populations in the full coupled model introduce a similar effect.

### 3.1 There Are no Globally Stable Limit Cycles when $w_{ss}=0$

When there is no self-excitation within the STN (i.e. $w_{ss}=0$) then it can be seen from the equations of the isolated channel system that there cannot be a globally stable limit cycle. Under these conditions, the Jacobian matrix at any fixed point has a negative trace and positive determinant, therefore, the fixed point must be stable. Let $q={\left(1+\exp(a_{j}\theta_{j})\right)}^{-1}$ (i.e. the constant term in Eq. (5)) and consider the box in phase space bounded by x=−q, y=−q, x=1−q, y=1−q; note that in general *q* is very small and so this box covers almost all of the phase space. It can be seen that the vector field around the edges of the box must point inwards. The box must therefore contain just one fixed point, which is stable. This means that globally stable oscillations are not possible. This analysis does not rule out the existence of pairs of stable and unstable limit cycles surrounding the fixed point, however, and so we will use qualitative analysis to investigate this possibility.

### 3.2 An Isolated Channel Cannot Oscillate Under Healthy Conditions

**Fig. 2:**
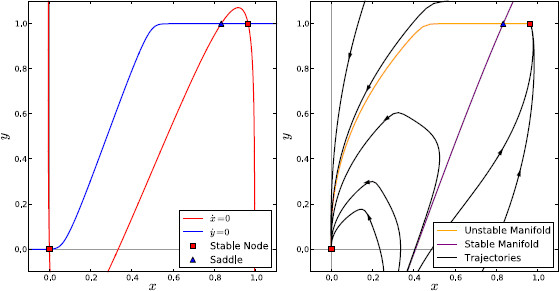
Isolated channel phase space under healthy conditions. Behaviour of the isolated channel system under healthy conditions with $w_{ss}=3.4$, I=0. *Left*: The nullclines and fixed points of the system. *Right*: Fixed points, stable and unstable manifolds of the saddle point, and example trajectories

The dynamics of the system when the healthy set of values for the fixed parameters were used can be understood qualitatively by examining the stability of the system’s fixed points and the shape of its nullclines for different values of the bifurcation parameters. Figure 2 shows the nullclines for a particular pair of values for $w_{ss}$ and *I*. With these parameters, the system is bi-stable, such that all trajectories in state space tend toward either a high or low level of activity in both nuclei depending on initial conditions. Also shown in Fig. 2 are the stable and unstable manifolds of the saddle point. Trajectories cannot cross these manifolds and the stable fixed point that any given trajectory tends toward depends on which side of the stable manifold its initial conditions lie upon. 

Adjusting the two parameters changes the ${\dot{x}}_{s}=0$ nullcline (the red line in Fig. 2): increasing $w_{ss}$ makes the slope of the middle branch steeper, while increasing *I* shifts the nullcline upward. Both of these changes increase the proportion of initial conditions that give trajectories tending to the high activity state, as would be expected from increased STN self-excitation or cortical input. If the parameters are raised past a critical point, the system undergoes a saddle-node bifurcation whereby the low activity stable fixed point and the saddle meet and annihilate, leaving the high activity state as the only fixed point of the system. Alternatively, if the parameters are lowered past a critical point then the high activity stable state disappears in the saddle-node bifurcation instead, leaving only the low activity state. Since these saddle-node bifurcations are the only bifurcations that the system undergoes, there is no possibility for limit cycles to arise when using the healthy fixed parameter values. The system displays hysteresis because increasing the parameter passes a critical value can cause trajectories to “jump” from one stable point to another, and reducing the parameter back past this critical value does not cause a jump back to the original fixed point.

### 3.3 Oscillatory Regimes Are Possible in Isolated Channels Under Parkinsonian Conditions

Applying the qualitative methods in the previous section to the system using the Parkinsonian set of fixed parameter values revealed a much richer array of possible dynamics and also suggested a parameter range within which bifurcations could be present. To fully understand the different dynamical regimes, numerical continuation was used. Continuation was first performed in one dimension by starting at a fixed point and varying a single parameter and then in two dimensions by starting at a bifurcation point and allowing both parameters to change.

**Fig. 3:**
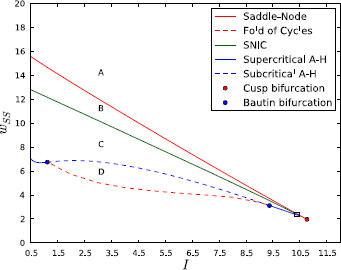
2D bifurcation diagram for isolated channel under Parkinsonian conditions. 2D bifurcation diagram showing the bifurcations that the isolated channel system undergoes under variation of *I* and $w_{ss}$ in the Parkinsonian case. A zoom of the area inside *the small rectangle in the lower right-hand corner* is shown in Fig. 4

**Fig. 4:**
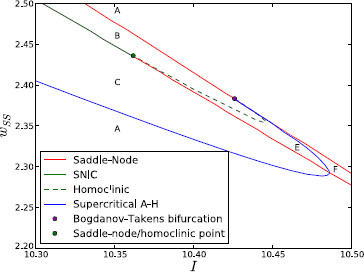
2D bifurcation diagram for isolated channel under Parkinsonian conditions (zoom). Zoom of the part of the diagram inside the black rectangle in Fig. 3

Figures 3 and 4 show the complete 2D bifurcation diagram of the system under Parkinsonian conditions. 

**Fig. 5:**
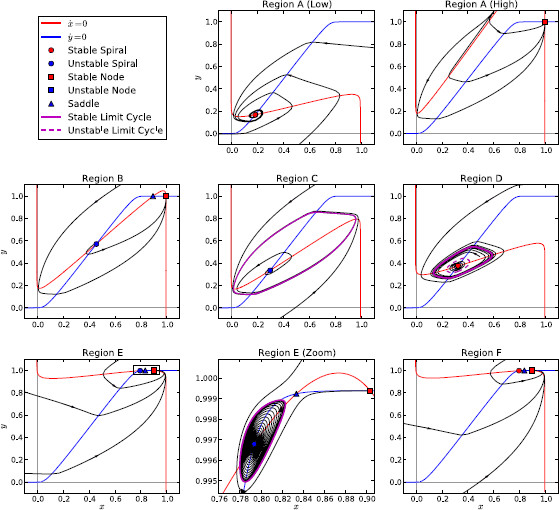
Phase portraits of isolated channel system under Parkinsonian conditions. Example phase portraits showing the behaviour of the isolated channel system within each of the regions of parameter space

The bifurcation curves divide the parameter space up into six regions. Within each region the phase portraits of the system are topologically equivalent, having the same number of stable and unstable fixed points and limit cycles. The characteristics of these features (such as frequency and amplitude of oscillation) may vary within regions. Figure 5 shows example phase portraits that are representative of the system’s behaviour in each of the regions. The parameters corresponding to each region in the figure are given in Table 2. 

**Table 2:** The parameter values that were used for each of the regions in Fig. 5

Region	*I*	$w_{ss}$
A (low)	2	4
A (high)	2	18
B	2	11.8
C	2	9
D	3.5	5
E	10.45	2.345
F	10.495	2.29

Region A makes up the majority of the parameter space. Within this region, the system possesses a single, stable, fixed point. The location of this fixed point in both dimensions increases with *I* and $w_{ss}$, as is expected from increased external stimulation or self-excitation. The behaviour of the system is more interesting in the other five regions (B–F), which together make up the large wedge-shaped area in the middle of the bifurcation diagram.

As one or both of the parameters is reduced from values that give a constant high rate of firing (the area above the wedge in the bifurcation diagram), they move toward and eventually pass through the saddle-node bifurcation curve and into region B. Two additional fixed points appear at this point, both unstable. Although in region B all trajectories still tend to the single stable fixed point, the effects of the saddle point’s manifolds causes some trajectories to take long paths around the phase space first. At the point where the parameters cross the saddle-node on invariant circle (SNIC) bifurcation curve, the stable node and the saddle point join together and the stable and unstable manifolds of the saddle point form a loop (a homoclinic orbit). Beyond the bifurcation, in region C, the saddle and the stable node have disappeared leaving the unstable spiral as the only fixed point. The homoclinic orbit has now become a stable limit cycle and so in this region all trajectories are attracted to the limit cycle and the system displays robust oscillations no matter what the initial conditions.

**Fig. 6:**
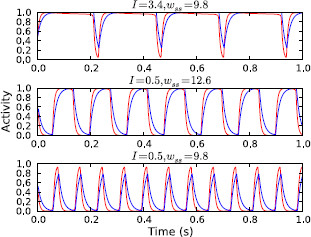
Oscillatory population activity in isolated channel model. Population activity over time for three points in region C, showing periodic pauses (*top*), bursts of high activity (*bottom*) and roughly even oscillation between high and low activity (*middle*). As in previous figures, the *red* and *blue lines* represent the activity of the STN (x(t)) and GPe (y(t)), respectively

Both the frequency and amplitude of the stable oscillations in region C vary as the parameters move around within it. Close to the SNIC bifurcation line the frequency is extremely low, since the effects of the “ghost” saddle point cause trajectories to pass very slowly through the part of the limit cycle that is close to where the saddle was located.^a^ When the parameters are within region C the activity of the sub-populations may show either low activity with short pulses of high activity, or the opposite, or something in between. Figure 6 illustrates this by showing a number of plots of population activity against time from within region C. 

The lower border of region C is, for the most part, an Andronov–Hopf bifurcation curve. This curve is divided into three segments—two supercritical parts that are separated by a long subcritical A-H curve. The points where the criticality of the bifurcation changes are the co-dimension-2 Bautin bifurcation points. The change in behaviour of the system as its parameters pass through the lower border of region C depends on whether they cross a sub- or super-critical A-H curve. In the case of two supercritical curves, this change is simple: the limit cycle shrinks around the unstable spiral until, at the bifurcation point, its amplitude becomes zero. At this point, the limit cycle disappears and the spiral becomes stable: the system has returned to region A.

**Fig. 7:**
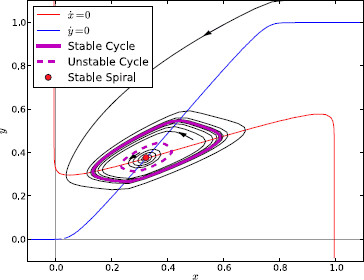
Phase portrait of Parkinsonian region D. Enlarged portion of Fig. 5 showing the nullclines and phase portrait of the isolated channel system in region D

The situation when the system leaves region C across the subcritical A-H curve is more interesting. In this case, the spiral becomes stable *before* the limit cycle has shrunk to zero amplitude. An expanded phase portrait of the system in this region is shown in Fig. 7. Since both the stable fixed point and the stable limit cycle have local basins of attraction, the region inside the stable limit cycle is divided into two concentric areas. Trajectories that begin within the inner area tend to the fixed point and trajectories that start within the outer area tend to the stable cycle. The border between these two areas is a new unstable limit cycle that appears at the point of subcritical bifurcation. The behaviour of the system within region D is therefore bi-stable and, depending on initial conditions, may show either steady-state or oscillatory activity levels. As the parameters move from the top to the bottom of region D, the stable limit cycle continues to shrink while the unstable limit cycle grows. The point at which the cycles meet and annihilate lies on the fold of cycles bifurcation curve. This leaves just one stable fixed point, returning the system to region A. 

While regions A–D make up the majority of the parameter space, there are two small additional regions (shown in detail in Fig. 4). The point at which the A-H curve terminates on the saddle-node curve is a co-dimension-2 Bogdanov–Takens (B-T) bifurcation point. Due to the normal form of the B-T bifurcation, this point must also be one end of a homoclinic bifurcation curve. The other end of the homoclinic curve is also located on the saddle-node curve at the saddle-node/homoclinic point; here the two curves merge and the saddle-node curve becomes a SNIC curve. At the point where the parameters cross the homoclinic curve from region B (unstable spiral, saddle, stable node) to region E, the stable and unstable manifolds of the saddle form a closed loop with each other. Beyond this bifurcation point, the two manifolds have crossed one another and a stable limit cycle appears between the saddle’s unstable manifold and the unstable spiral (see pp. 185–190 in [50]). Like region D, region E has a bi-stability between steady-state and oscillatory behaviour that depends on initial conditions. The set of initial conditions that leads to oscillations is very small, however, due to the shape of the saddle point’s manifolds. 

If the system’s parameters leave region E through the supercritical A-H curve, then the unstable spiral becomes stable and the limit cycle is destroyed. Behaviour in this region (region F) is still bi-stable, but the two stable states are both fixed points so there can be no oscillation. Furthermore, these two states are extremely close to each other in phase space, both being regions of high activity. The parameters can leave region F through one of two parts of the saddle-node curve. Crossing either of these parts results in the loss of one of the stable fixed points and the saddle, leaving just one fixed point, which is stable.

**Fig. 8:**
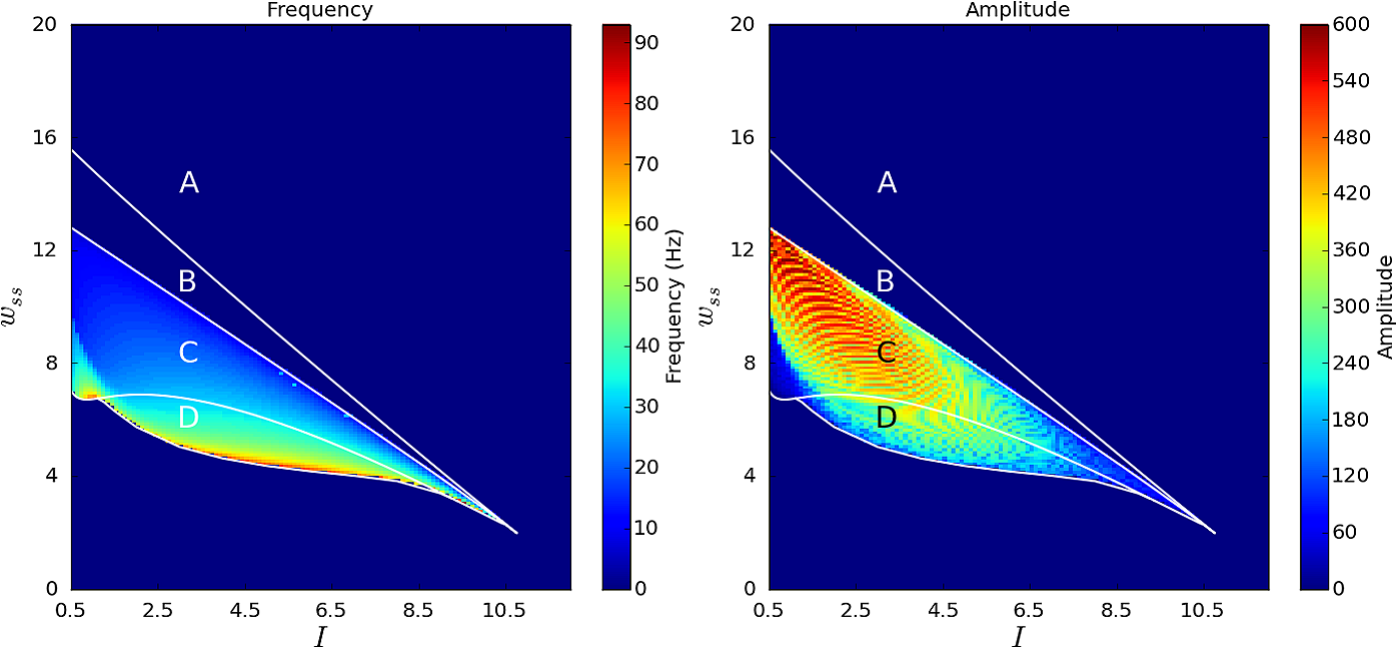
Effect of parameters on oscillation frequency and amplitude in an isolated channel. The frequency and amplitude of the strongest oscillation present for each pair of parameter values for the isolated channel system under Parkinsonian conditions. The bifurcation curves and regions are shown for ease of comparison with Fig. 3

Since bifurcation analysis revealed a number of oscillatory regions in the parameter space a further numerical experiment was performed to investigate the characteristics of these regions. Specifically, a large scale set of numerical simulations were performed to determine how the frequency and amplitude of the limit cycles varied with the parameters. The parameter space was divided up into a uniform grid and, for each pair of parameter values, the system was simulated for a period of time. The power spectrum of the resulting activity was computed using a fast Fourier transform (FFT) and the frequency of the strongest oscillation visualised. Figure 8 shows the results of these computations. The same simulations were performed using the healthy fixed parameters, but as expected no oscillations were seen and so the results are not shown. 

These results are what would be expected based on the bifurcation analysis. Only regions C and D contain oscillatory activity (the only other oscillatory area in Fig. 4, region E, is too small to be shown here). The frequency of oscillations decreases to zero as the parameters move toward the SNIC bifurcation curve (the boundary between regions B and C) and increases as the parameters are decreased away from this curve. As previously discussed, the amplitude of the oscillations is greater when the parameters are close to the SNIC bifurcation line, since the unstable spiral and “ghost” saddle point are far apart here. The frequency in much of region C is in the *β* band, but region D contains some areas of higher frequency oscillation (up to about 50 Hz, which falls within the low part of what is termed the *γ* band). These frequencies can only be considered as some approximation of rhythms that would be found in the real STN-GPe network.

We will now use the results of our analysis of the isolated channel model to investigate how the dynamics of the system change as coupling between channels is introduced.

## 4 Analysis of the Coupled Channels Model

The coupled channels model is a 2*N* dimensional system (with N>1), which means that analysis is much more difficult than for the isolated channel model. We will begin by discussing the parameter values that were selected before presenting some general results that show that the coupled channels model has an oscillatory regime that is very robust and exists for a wide range of parameters. Finally, Sect. 4.3 will briefly describe the detailed structure of the attractors that the system has.

### 4.1 Parameter Choice

When studying the coupled channels model, we used the same values for the fixed parameters as were used in the isolated channel model (see Table 1). As before, the connection strengths were divided into a healthy set and a Parkinsonian set. However, since there is no known mechanism whereby STN neurons can excite other STN neurons, we chose to fix $w_{ss}=0$. Although the analysis of the isolated channel model found $w_{ss}>0$ to be a necessary condition for oscillations, we hypothesised that the coupled channels model might be able to oscillate with $w_{ss}=0$, since the path from an STN subpopulation to its neighbouring STN subpopulation and back again will have the effect of indirect delayed self-excitation. The coupled channels model has an additional parameter that can be varied (*α*), which controls the strength of inhibition between neighbouring GPe sub-populations as a proportion of the self-inhibition within GPe sub-populations ($w_{gg}$). Since we are basing our model on the idea that sensorimotor channels remain largely segregated throughout the STN/GPe network [3,33,34], we argue that α<1 is the physiological range for this parameter. We studied the system under variation of *α* and *I*, using the results of our analysis of the isolated channel model to guide the selection of a reasonable range of values for *I*.

### 4.2 Oscillations Require Strong Coupling, Particularly Under Healthy Conditions

We began by manually carrying out many numerical simulations of different coupled systems, varying the number of channels, connection topology, *α* and *I*, and whether the healthy or Parkinsonian fixed connection strengths were used. Each simulation was started from random initial conditions. During this experimental work, we found that for relatively low lateral coupling (e.g. when α<0.5) the systems always converged to a single fixed-point attractor. None of our experiments with low *α* found oscillatory regimes or multi-stability. We found that it is possible (for some values of *I*) for the fixed-point attractor to undergo a supercritical Andronov–Hopf bifurcation as the parameter *α* is increased toward 1. This bifurcation causes a stable limit cycle of small amplitude to appear. This limit cycle is a global attractor. The range of values of *I* for which this bifurcation exists depends upon whether the healthy or Parkinsonian connection strengths are used: It is much wider in the Parkinsonian case than in the healthy case.

**Fig. 9:**
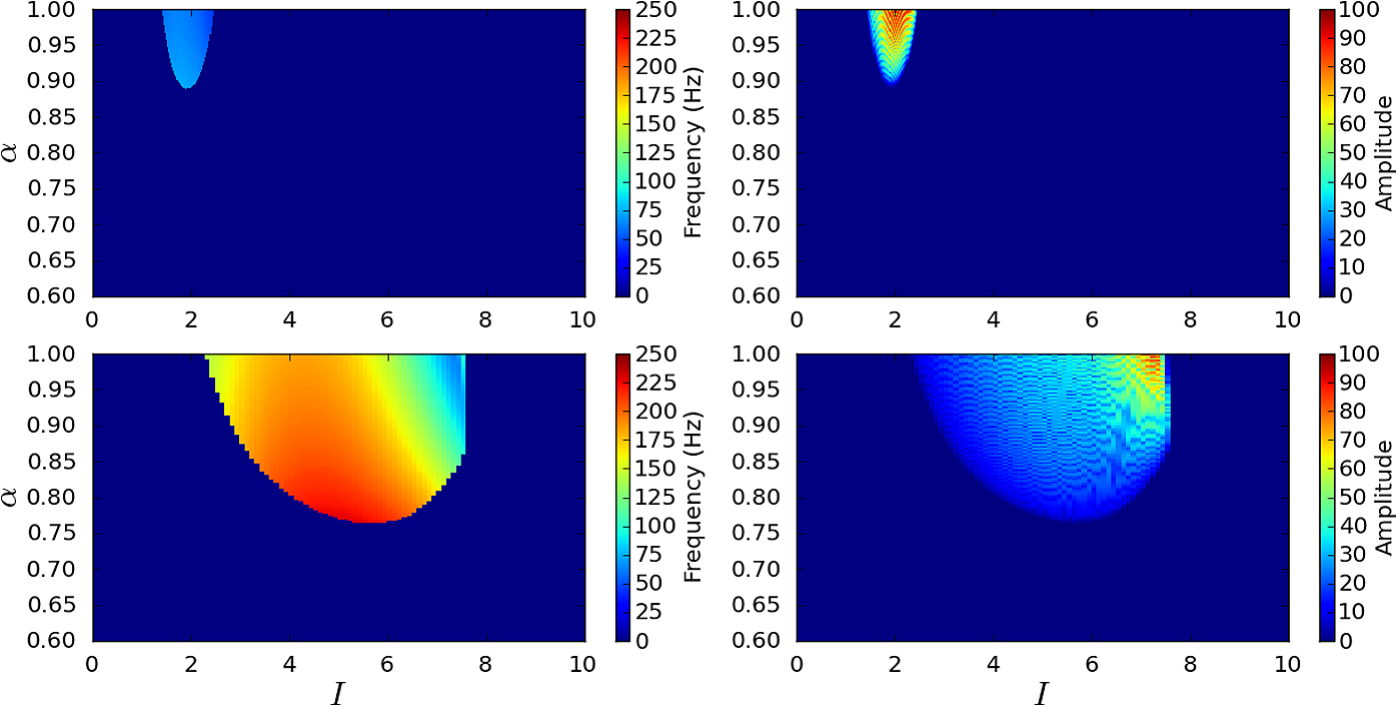
Effect of parameters on oscillation frequency and amplitude in coupled channels. The frequency (*left*) and amplitude (*right*) of the strongest FFT bin encountered during numerical simulation from random initial conditions across a range of parameter values. *The top row* shows the system under healthy conditions and *the bottom row* shows Parkinsonian conditions. The system here has 5 channels arranged in a line topology

To make this investigation more rigorous, we ran similar large-scale simulations to the one which was used to generate Fig. 8, for a range of different connection topologies and channel counts. Figure 9 shows one such result for 5 channels coupled in a line topology, under both healthy and Parkinsonian conditions. It can be seen that this appears to confirm our finding that oscillations require reasonably strong lateral coupling and are much more prevalent under Parkinsonian conditions. This fact appears to be generally true regardless of the connection topology used or number of channels (up to 100 channels were used). 

**Fig. 10:**
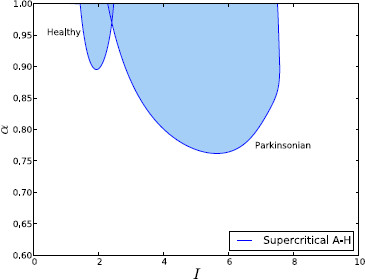
Coupled channel bifurcation diagram. Continuation of the supercritical A-H in the (I,α) parameter plane under both healthy and Parkinsonian conditions. *The shaded areas* correspond to oscillatory activity. The system here has five channels arranged on a line

To further confirm these results, we used numerical continuation software to plot the curve of the A-H bifurcation in parameter space. Figure 10 shows the results of these computations for both the healthy and Parkinsonian cases, using five channels arranged on a line. In the case of five channels on a circle, the bifurcation is more complex because a symmetry means that two pairs of complex conjugate eigenvalues simultaneously cross the imaginary axis (Hopf–Hopf bifurcation with equal pairs of eigenvalues). 

### 4.3 Detailed Attractor Structure Depends on Channel Count and Topology

Qualitative investigation of the coupled channels system revealed that the attractors of each system are structured in a way that depends on the coupling topography (i.e. circle or line) and whether the number of channels was odd or even. This section will briefly illustrate the different attractor structures that our model can have in order to demonstrate the range of possibilities.

**Fig. 11:**
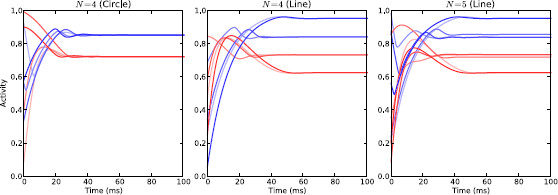
Steady state activity of coupled channels model. Steady-state activity of the healthy coupled channels model with α=0.5 and I=2.5 under three different configurations. The activity of the STN sub-populations is shown in *shades of red* and the activity of the GPe sub-populations is shown in *shades of blue*. *Left*: circular topology with four channels (same activity level across all channels); *middle*: line topology with four channels (pairs of channels with same activity); *right*: line topology with five channels (pairs of channels with same activity plus middle channel)

We first consider the effect of gradually raising the value of *α* up from zero while keeping *I* constant. When α=0, we know from analysis of the isolated channel model that all of the STN sub-populations will converge to some fixed activity level (determined by *I*) and all the GPe sub-populations will converge to some other fixed level (i.e. there is a single fixed point where $x_{1}=x_{2}=\cdots =x_{N}$ and $y_{1}=y_{2}=\cdots =y_{N}$). Increasing *α* changes the co-ordinates of this single steady-state in phase space in a way that depends on whether the system is coupled as a line or a circle. In the case of channels arranged on a circle, there continues to be a single activity level for all STN sub-populations and another level for GPe sub-populations, but increasing *α* decreases the GPe level and increases the STN one. When the channels are arranged on the line, their steady-state activity levels become paired symmetrically (i.e. $\left(x_{i},y_{i})=(x_{N-(i-1)},y_{N-(i-1)}\right)$). When *N* is odd, the centre channel has its own unique activity level. Increasing *α* causes the activity levels associated with the different channel pairs to spread out in phase space. Figure 11 shows the steady-state activity for a number of topologies (circle, line with *N* even, line with *N* odd). 

**Fig. 12:**
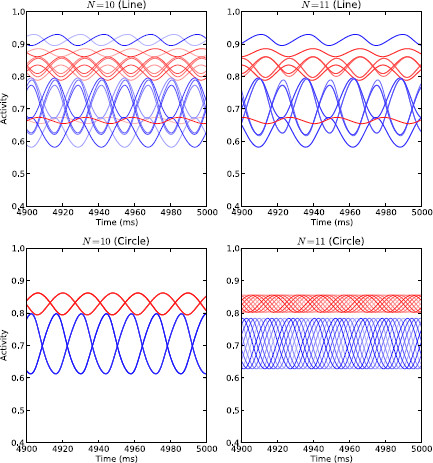
Oscillatory activity of coupled channels model. Oscillatory activity of the coupled channels model with α=0.95, I=2.5 and healthy connection strengths, under four different configurations. The activity of the STN sub-populations is shown in *shades of red* and the activity of the GPe sub-populations is shown in *shades of blue*. *Top left*: line topology with ten channels (five anti-phase pairs); *top right*: line topology with eleven channels (five in-phase pairs plus middle channel); *bottom left*: circle topology with ten channels (two anti-phase groups); *bottom right*: circle topology with eleven channels (splay state)

The system begins to oscillate when *α* passes some critical value $\alpha_{\mathrm{crit}}$. The precise value of $\alpha_{\mathrm{crit}}$ depends on *I*, *N* and the coupling topography/strengths, but in every case the stable attractor becomes unstable and a new stable oscillatory attractor appears. The amplitude of the associated oscillations is small near the bifurcation and increases as *α* moves further away from its critical value. The oscillatory activity can take four different forms depending on the coupling topography and whether *N* is odd or even. In the case of the line topography, each of the pairs of channels begin oscillating together either anti-phase (*N* even) or in-phase (*N* odd). With the circle topography, the channels all oscillate identically, but in either 2 anti-phase groups (*N* even) or in a “splay state” with a constant phase-shift between channels such that they span the oscillation period (*N* odd). Additionally, for the circle with *N* odd, it appears that additional bifurcations can occur as *α* is increased further that result in additional stable oscillatory attractors besides the splay state. Figure 12 shows the four main patterns of oscillatory activity. 

**Fig. 13:**
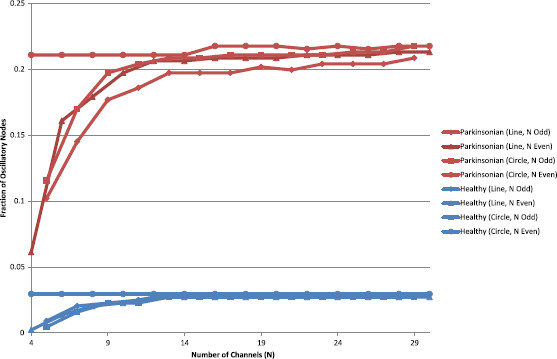
Area of oscillatory region in parameter space. The area of the oscillatory region in diagrams similar to those in Fig. 9, for different values of *N* and coupling topographies. In every case, the area is much larger under Parkinsonian conditions than healthy ones and is not significantly affected by *N* or the coupling topography, for values of *N* greater than approximately 14

In order to confirm that the general behaviour of the system was independent of *N* and coupling topography, we generated diagrams similar to Fig. 9 for values of *N* from 3 to 30, under both coupling schemes. Qualititative inspection showed that all the diagrams were similar, as expected. For a more objective measure, we computed, from each diagram: the fraction of nodes in the (I,α) parameter grid that gave oscillatory activity, the minimum coupling strength (*α*) that gave oscillations, and the average frequency of oscillation. These calculations confirmed that oscillations are present for a much greater range of parameter values under Parkinsonian conditions (Fig. 13), and similarly that the minimum value of *α* required for oscillations was always much higher in the healthy case than in the Parkinsonian one (0.8 vs. 0.65, not shown). As expected, these measures tended to a constant level (which did not vary with coupling topography) as *N* was increased, showing that the general behaviour (oscillatory versus steady-state) was independent of channel count and topography. The calculations also found that the average oscillation frequency did not vary much with *N* or coupling topology, but that this average frequency was consistently much lower under healthy conditions than Parkinsonian ones (55 Hz vs. 130 Hz, not shown). 

## 5 Discussion

### 5.1 The STN and GPe May Generate Oscillations when Lateral Coupling Is Strong

The analysis of the coupled channels model above demonstrates that the range of parameters that cause oscillations in a system of *N* STN/GPe subpopulation pairs, laterally coupled at the GPe level, is relatively independent of the coupling topology used and the value of *N* (as long as N>3). In all cases, the sub-populations all tend to a constant level of activity when the strength of lateral inhibition is weak compared to inhibition within GPe sub-populations. When lateral inhibition is made almost as strong as the inhibition within GPe sub-populations then the network as a whole can begin to generate oscillations when the level of cortical input received by each channel is within a certain range; this range is much wider when the remaining connection strengths are set at values representing the Parkinsonian basal ganglia.

There is some experimental evidence that suggests that this result could represent what happens in the real basal ganglia. LFPs recorded simultaneously from multiple sites within the rat globus pallidus (homologous to the human GPe) display a degree of coherence that varies with global brain state: Under anaesthetised slow wave activity (SWA) conditions, the LFP signals have little coherence, but when the brain state becomes “globally activated” the signals become much more coherent with one another suggesting an increased level of lateral coupling [51]. In terms of our model, this would correspond to the value of *α* varying with brain state—low during SWA and higher during global activation. Interestingly, a similar study using rats that were chronically dopamine depleted via 6-hydroxydopamine (6-OHDA) lesion found that the characteristic *β* LFP peak in the STN was present only in the globally activated brain state, not during SWA [52]. Our model suggests that this oscillatory activity may be generated locally by the STN/GPe circuit as a result of the increased lateral coupling between GPe sub-populations that is seen during global activation. 

The frequency of oscillations generated by our model is generally much higher than the 15–30 Hz *β* band—although it is interesting to note that the parameter values that resulted in the largest amplitude of oscillation were those that gave the lower frequency oscillations, including the *β* band (Fig. 9). Although we found that shifting the fixed connection strengths toward their healthy values reduced this average frequency (whilst shrinking the oscillatory region of parameter space), we did not find a simple relationship between the frequency of oscillation and any one individual connection strength. It is possible that more complex coupling topologies (for example, linking each GPe subpopulation with more of its neighbours, with a strength that decreased with distance) could have the effect of reducing oscillation frequency. Our definition of the frequency of oscillation was also very simple: we considered only the frequency of the highest peak that was found across the power spectra of all of the sub-populations’ activity. A more thorough study should examine the entire spectrum in each case and check for a peak at *β*, and could consider a measure that would more accurately correspond to a simulated LFP recording (such as the summation of activity across all channels). Finally, it is possible that the time constants that were used (particularly for the GPe sub-populations) were significantly different to the typical cell membrane time constants of the populations we are modelling. Experiments have reported a wide range of possible values for membrane properties of GPe neurons [53]. At present, our model only demonstrates that some oscillatory activity is possible in the Parkinsonian STN-GPe when the level of lateral coupling in the GPe is sufficiently strong. 

### 5.2 Individual Channels Are Capable of Complex Dynamics

Our analysis of the isolated channel model demonstrates that, when the Parkinsonian connection strengths are used, a simple model of a coupled pair of STN and GPe sub-populations can generate dynamic behaviour that is either steady-state (regions A and B), oscillatory (region C), or bi-stable between a steady and oscillating state (region D). The oscillatory and bi-stable regimes rely on a non-zero degree of STN self-excitation. This section will describe one possible model of basal ganglia movement processing that these dynamics could represent. Here, we do not mention regions E and F as they are extremely small and are therefore unlikely to correlate with observed features of basal ganglia (dys)function.

We consider a system that consists of multiple isolated channels which all have parameters such that they are in region D (see Fig. 7). Each channel can be switched between oscillation and steady-state activity by a short transient external perturbation of the activity in either STN or GPe. To take a channel from steady-state to oscillatory activity, this perturbation must be sufficient to move the system outside the basin of attraction of the fixed point (this is the region enclosed by the unstable limit cycle). Transferring the system to the steady-state is more difficult. The perturbation must arrive at the correct time in the oscillatory cycle in order to move the current position in phase space toward the unstable cycle. The correct time depends on whether the short external perturbation affects the STN or GPe, and whether it has an excitatory or inhibitory effect. For example, an inhibitory perturbation applied to a GPe subpopulation must occur during the high activity phase of oscillation as this will move the trajectory down in phase space and, if the perturbation is of the correct amplitude, bring the trajectory inside of the unstable limit cycle where it will be attracted in to the stable spiral.

LFP recordings reveal a drop in synchronous *β* oscillations in the basal ganglia prior to and during movement [54] and, according to our interpretation, this corresponds to one or more channels transferring from a limit cycle to a stable fixed-point’s basin of attraction. This transfer requires precisely timed perturbation. One possible source for this perturbation is the inhibitory input that the GPe receives from the striatal medium spiny neurons (MSNs). This projection is organised in a segmented manner, which suggests that each of our channels receives striatal input from a different set of MSNs [55]. Recordings in monkeys have found that a sub-set of these neurons, the phasically active neurons (PANs), are normally silent but show short bursts of activity just prior to movement [35]. Simultaneous LFP and unit activity recordings from the striatum of healthy behaving monkeys reveals that there is a transient *β* rhythm in the striatal LFP and, furthermore, that the firing of PANs occurs at a particular point in the cycle of this oscillation [22]. If the striatal and pallidal *β* LFP oscillations are synchronised to some degree (this is currently unknown), then it is possible that the PAN bursts arrive during the correct part of the STN-GPe oscillation cycle to push a channel into the stable state. After a movement has been completed, the channel can easily be switched back to its *β* oscillatory mode by an excitatory or inhibitory perturbation of its STN or GPe sub-population. Each channel that is in region D therefore acts as a switch or filter. Assuming each channel corresponds to a movement or body region, synchronised oscillatory activity in the circuit prevents movement either by reducing information transfer or acting as a global “anti-kinetic” signal. When movement is required, precisely timed striatal input effectively switches the oscillations off temporarily.

If, due to some modulation of cortical input or STN self-excitation $\left(I,w_{ss}\right)$, the system moves close toward region C then the basin of attraction for the stable fixed point becomes smaller. When this happens, the external perturbation required to escape the oscillatory region must be of larger amplitude and timed more precisely. Finally, when the parameters pass into region C, the fixed point loses stability and no external perturbation of trajectories would be able to stop the system oscillating. We claim that these changes may correspond to the daily fluctuations in the severity of the hypo-kinetic motor symptoms of Parkinson’s disease, with region C corresponding to the akinetic state where movement cannot be initiated at all.

An alternative biological interpretation of the bifurcation diagram does not involve external perturbations, but instead relies on the fact that when the system is close to one of the bifurcation curves its behaviour depends very sensitively on the parameters. For example, close to the SNIC and fold cycle curves small changes in cortical input can switch the system between oscillatory and steady-state behaviour. Under Parkinsonian conditions where there is a large oscillatory region, a greater value of *I* may be needed to escape this region.

The physiological plausibility of this mechanism for activating and deactivating different movement channels is limited by the fact that the bi-stable region only exists when the Parkinsonian strengths are used and STN self-excitation is non-zero. However, our results have shown that introducing a degree of coupling between channels unlocks much more interesting dynamics within each channel, even in the healthy case. Further preliminary work (not shown here) suggests that introducing heterogeneity to the level of cortical input that each channel receives makes the possible dynamics richer still. It is possible that under these more realistic conditions, there are regions of parameter space where channels can exhibit similar bi-stable behaviour to what is described here.

Our analysis of the isolated channel model could help to identify parameter values that give interesting dynamics (such as oscillations and bi-stability) in the coupled model. A possible approach is to use numerical continuation to smoothly move the system from its oscillating (or bi-stable) isolated channel state (with non-zero $w_{ss}$) to a similar state with GPe coupling and $w_{ss}=0$. To do this, we could take $w_{ss}=(1-\alpha )w_{ss}^{\prime }$, where $w_{ss}^{\prime }$ is an STN self-excitation strength that was found to give oscillations or bi-stability in the isolated model. In this modified model, α=0 would correspond to isolated channels with STN self-excitation and α=1 would correspond to coupled channels without STN self-excitation. If our hypothesis that lateral inhibition between neighbouring GPe sub-populations has a similar effect to STN self-excitation is correct, it should be possible to examine what happens to the different dynamic regimes as one mechanism replaces the other.

### 5.3 Comparison with Other Models

The results of our analysis of a single isolated channel agrees, to a large extent, with the results of the study of Holgado et al. [32], which considered the entire STN and GPe each as single populations and from which our parameters were taken. As in [32], stable *β* oscillations occur only when the parameter values corresponding to the Parkinsonian state are used. The model presented here is simpler than that of [32] as it does not attempt to model the synaptic transmission delay between sub-populations. This simplicity made bifurcation analysis possible, which revealed a region of interesting behaviour that is bi-stable between oscillatory and steady-state activity. Such behaviour was not seen in the model presented in [32], presumably as it only occurs when the degree of STN self-excitation is non-zero and this was not the case in the model of Holgado et al. 

Another previous modelling study, by Gillies et al. [29], considered a population-level model of the STN-GPe circuit that is also very similar to our isolated channel model. They described three different states for the system: a single fixed point, an oscillatory state that showed low frequency short periods of high activity, and a state that was bi-stable between two stable fixed points. All of these states are also present in the model presented in this paper. The single fixed-point state corresponds to the system when healthy values of the fixed parameters are used or when the Parkinsonian values are used and the system is in region A. The oscillatory state corresponds to region C of the Parkinsonian parameter space. Finally, the parameter values that give bi-stability between two fixed points are found in region F. Gillies et al. hypothesised that this could represent the physiological mode of operation of the STN-GPe circuit, but our model suggests that this is unlikely as region F represents an extremely small part of the parameter space. This means that the fixed-point bi-stable state is very fragile and small changes in cortical input would move the system out of it. Furthermore, within region F the two stable fixed points are very close together in phase space and so the bi-stability would only switch between two very similar levels of activity. Instead, our model suggests that the physiological state is in fact bi-stable between a fixed point and a limit cycle. 

Berns and Sejnowski developed a population-level model of action selection in the basal ganglia that embodies the idea of multiple sensorimotor pathways [56]. Each channel in this model contains sub-populations for the cortex, striatum, GPi/e, and thalamus; however, the STN is modelled as a single global sub-population that is the only link between channels. The authors consider how Parkinsonian conditions affect the ability of the model to select actions, but they do not investigate its ability to generate oscillatory behaviour in this case. This model does not contain the projection from STN back to GPe, and so cannot be used to study the possible pacemaker role of this circuit. A very similar model by Gurney et al. [57,58] also considers the effect of dopamine depletion in terms of the failure of action selection and again does not examine the possibility of oscillations emerging. A more refined version of this model that used the same functional connectivity but with computational current-based modelling of the individual neurons within each sensorimotor channel exhibits several features that are found in experimental recordings under both healthy and Parkinsonian conditions [59], including oscillations (although in this case only the *γ* band is considered). Since the mathematical complexity of this model is much greater than population-based models, mathematical analysis (such as considering the dynamical capabilities of individual channels) becomes intractable.

In our model, the strength of GPe self-inhibition ($w_{gg}$) is increased under Parkinsonian conditions. In contrast, some models (notably that of Terman et al. [28]) find that a reduction in pallidal self-inhibition may facilitate increased rhythmic activity in the STN-GPe network. There is some evidence to suggest that this decrease of $w_{gg}$ in the Parkinsonian case is more appropriate, based on the effects of increased striatal-pallidal activity on GABA release in the GPe [60,61]. It would be interesting to see how our results would differ with decreased $w_{gg}$ under Parkinsonian conditions.

### 5.4 Further Work

The models described in the previous section raise the interesting question of whether or not our model is capable of performing action selection. When analysing the dynamics of an individual channel in Sect. 3.3, we found a hypothetical mechanism by which channels could be switched on and off and this could form part of a system for action selection. As a result of its symmetry our coupled channels model could only produce dynamics that were common across all channels, which is clearly not useful for action selection, and so the first step will be to break this symmetry. One way to do this is to provide a heterogeneous level of cortical input to each STN subpopulation.

It may also be possible to use this model to investigate the basis for the remarkable improvement in symptoms that can be achieved through high-frequency electrical stimulation of the STN [62]. One potential way to incorporate the effects of deep-brain stimulation (DBS) into the model is to add an external periodic input to the equation for activity in one or more STN sub-populations. When investigating the isolated channel model, we observed that with parameters set such that it is in a region with stable *β* oscillations, there exists a range of frequencies for the external input that cause the oscillations to become chaotic, flattening the power spectrum. This range of frequencies appears similar to the range of clinically effective DBS frequencies. This interesting result requires further investigation.

This paper is based on the assumption that excessive *β* activity plays a causative role in the hypo-kinetic symptoms of Parkinson’s disease, but some evidence suggests that it is merely a correlative epiphenomenon. When the progression of Parkinson’s disease is simulated in monkeys by the selective lesioning of dopaminergic SNc neurons over the course of many days, oscillatory activity is not observed in the firing rate of individual GPi neurons until long after motor symptoms have appeared [63]. It is not clear, however, whether or not LFP signals (where the *β* peak is usually seen) in the GPi are related to unit activity in that nucleus [9]. Other studies with rats have compared the effects of chronic SNc lesioning with acute dopamine blockade and found that only the chronic condition results in a peak in *β* power in STN LFP [52] and motor cortex ECoG [64], even though both chronic and acute dopamine depletion/blockade induce akinesia. Such evidence does not necessarily rule out the possibility of *β* oscillations having an anti-kinetic effect, however, since acute dopamine blockade may disrupt motor pathways in a way which is different to the mechanism by which *β* oscillations act to prevent movement. Even if excessive *β* activity is simply a side-effect of chronic loss of dopaminergic input to the basal ganglia that does not directly cause Parkinsonian motor symptoms it may still serve as a marker for this neuronal damage that is useful experimentally [13]. Furthermore, it has been proposed that elevated *β* LFP power could be used as a trigger for a new generation of “on-demand” devices for DBS [65,66]. Whether the relationship between abnormal *β* synchronisation and the hypokinetic symptoms of Parkinson’s disease is causative or merely correlative, it is clearly a significant characteristic of the Parkinsonian basal ganglia that should be properly understood. Further modelling work will help to achieve this understanding.

## Appendix

**Video 1:**http://www.youtube.com/watch?v=sT0_Ognnsns

This video shows the activity in a single pair of coupled STN-GPe subpopulations under Parkinsonian conditions. Each particle is a 2-dimensional vector representing a point in state space and the particles are initialized to have random positions. As the simulation runs, each particle’s position evolves according to the equations of the system in either forward time (red particles) or backward time (blue particles). Each frame a random sub-set of particles are reset to a new random position. The level of STN self-excitation ($w_{ss}$) is gradually increased, showing a range of dynamical regimes: globally single stable fixed point, bistability between fixed point and stable oscillations, globally stable oscillations, and back to a globally stable fixed point.

**Video 2:**http://www.youtube.com/watch?v=C-h-BBb9D9M

The first part of this video shows the activity of five parallel channels under Parkinsonian conditions, each of which is made up of a coupled STN-GPe subpopulation pair. As in Video 1, many sets of initial conditions are chosen uniformly from across the 10-dimensional phase space and each set of initial conditions is integrated in parallel using the computer’s graphical processing unit (GPU), with random resetting. For each set of initial conditions being integrated, the level of STN and GPe activity is projected onto a different part of the screen (and in a different colour) for each channel. A white line is used to link the particles corresponding to the first set of initial conditions (which are never randomly reset). As the strength of coupling between the channels (*α*) is increased, oscillatory activity appears that is anti-phase between neighbouring channels.

The second part of this video is similar to the first part except that 799 channels are shown. Only one set of initial conditions is used here and there is no resetting. The vertical position of each dot indicates the level of GPe activity in each channel. As before, oscillations appear as lateral coupling is increased and the shape of the oscillatory attractor appears to be very non-regular.

In both parts of the video the level of cortical input is fixed across all channels at 5.0.

## Competing Interests

The authors declare that they have no competing interests.

## Authors’ Contributions

RM, NY, FN and RB designed the study, performed the experiments, and analysed the results. OB assisted with simulation and bifurcation analysis of the multiple channels model. All authors contributed to the writing of the manuscript. All authors read and approved the final manuscript.
